# Combined Impact of Canada Goldenrod Invasion and Soil Microplastic Contamination on Seed Germination and Root Development of Wheat: Evaluating the Legacy of Toxicity

**DOI:** 10.3390/plants14020181

**Published:** 2025-01-10

**Authors:** Guanlin Li, Yi Tang, Hongliang Xie, Babar Iqbal, Yanjiao Wang, Ke Dong, Xin Zhao, Hyun-Jun Kim, Daolin Du, Chunwang Xiao

**Affiliations:** 1 School of Environment and Safety Engineering, Jiangsu University, Zhenjiang 212013, China; liguanlin@ujs.edu.cn (G.L.); ty12212021@163.com (Y.T.); hongliangxie1018@163.com (H.X.); 17797313436@163.com (Y.W.); 2 Jiangsu Collaborative Innovation Center of Technology and Material of Water Treatment, Suzhou University of Science and Technology, Suzhou 215009, China; 3 School of Ecology and Environment, Inner Mongolia University, Hohhot 010021, China; 4 Division of Bio Convergence, Kyonggi University, Suwon 16227, Republic of Korea; 5 Department of Civil and Environmental Engineering, College of Engineering, Seoul National University, Seoul 08826, Republic of Korea; zhaoxin@snu.ac.kr; 6 Department of Forest Resources, Chonnam National University, Gwangju 61186, Republic of Korea; 7 Jingjiang College, Institute of Environment and Ecology, School of Environment and Safety Engineering, School of Emergency Management, School of Agricultural Engineering, Jiangsu University, Zhenjiang 212013, China; ddl@ujs.edu.cn; 8 College of Life and Environmental Sciences, Minzu University of China, Beijing 100081, China

**Keywords:** emerging contaminants, microplastics, alien plant invasion, combined effect, seed germination, root development

## Abstract

The concurrent environmental challenges of invasive species and soil microplastic contamination increasingly affect agricultural ecosystems, yet their combined effects remain underexplored. This study investigates the interactive impact of the legacy effects of Canada goldenrod (*Solidago canadensis* L.) invasion and soil microplastic contamination on wheat (*Triticum aestivum* L.) seed germination and root development. We measured wheat seed germination and root growth parameters by utilizing a controlled potted experiment with four treatments (control, *S. canadensis* legacy, microplastics, and combined treatment). The results revealed that the legacy effects of *S. canadensis* and microplastic contamination affected wheat seed germination. The effects of different treatments on wheat seedling properties generally followed an “individual treatment enhances, and combined treatment suppresses” pattern, except for root biomass. Specifically, the individual treatment promoted wheat seedling development. However, combined treatment significantly suppressed root development, decreasing total root length and surface area by 23.85% and 31.86%, respectively. These findings demonstrate that while individual treatments may promote root development, their combined effects are detrimental, indicating a complex interaction between these two environmental stressors. The study highlights the need for integrated soil management strategies to mitigate the combined impacts of invasive species and microplastic contamination on crop productivity and ecosystem health.

## 1. Introduction

Microplastics, defined as plastic particles smaller than 5 mm, have become a pervasive environmental pollutant, increasingly recognized for their long-term persistence and ecological impact [1,2,3]. Due to their small size and physical-chemical properties, these particles are difficult to remove from the environment. Microplastics are characterized by strong hydrophobicity, chemical stability, and the ability to adsorb environmental pollutants, such as heavy metals and organic contaminants [4]. Thus, microplastics in soil pose toxic threats to soil biota and plants, leading to ecological hazards, bioaccumulation, and potential risks to human health through the food chain [5].

In terrestrial ecosystems, especially in agricultural soils, microplastics accumulate through several pathways, including the fragmentation of agricultural plastic mulches, the application of sewage sludge and compost, and atmospheric deposition [6]. Their widespread presence in soils, particularly in farming areas, raises concerns about their potential impact on soil health and crop productivity. However, studies investigating the effects of microplastics on agricultural soil and crops have yielded inconsistent results. Some studies suggest that microplastics may not immediately impair crop growth, particularly when concentrations are low [7,8,9]. In contrast, long-term residue or high concentrations of microplastics have been shown to negatively influence the physiological and biochemical characteristics of plants, such as root system architecture, nutrient absorption, and biomass production [10,11]. Additionally, soil microplastics can alter soil characteristics, such as porosity and water retention capacity, which may disrupt the delicate balance of soil–plant interactions and reduce overall soil fertility [12,13]. Moreover, the legacy effects of microplastics in the soil create a novel environment that promotes invasive species, and in the end, may help explain the competitive success of species of an invasive character in novel environments [13]. As microplastic pollution in agricultural soils worsens globally, it poses increasing risks to agriculture and human health, demanding further attention and research. Similarly, a previous researcher has demonstrated that microplastics have a legacy effect by affecting biomass and root growth traits, highlighting their key role in plant–soil feedback [14].

The perennial plant known as Canada goldenrod (*Solidago canadensis* L., hereafter *S. canadensis*) is native to North America and belongs to the Asteraceae family. The species is characterized by its rapid reproductive capacity, efficient germination, and continuous growth, making it highly adaptable and resilient [15]. Simultaneously, the invasion of alien plant species, such as *S. canadensis*, poses another serious environmental challenge, especially within agricultural ecosystems. Invasive species are known to significantly disrupt native plant communities and soil ecosystems through their aggressive growth and competitive resource acquisition [16,17]. By releasing allelopathic chemicals, invasive plants can alter the soil’s abiotic environment and biotic communities, with these chemicals often lingering in the soil long after the plants have been removed [18,19,20,21,22]. This lasting impact, referred to as a “legacy effect”, can persist for months or even years, influencing the growth and development of subsequent plants by modifying soil conditions and microbial communities [23,24]. There is substantial evidence that *S. canadensis* invasion inhibited the biomass of alfalfa, barley, and carrots due to the high soil ferulic acid concentration, evidenced by the allelochemical secretion in the soil by affecting diverse plant species [25,26]. These legacy effects often create an environment that favors invasive species over native ones, allowing the former to dominate ecosystems and further suppress agricultural productivity.

Studies on the effects of microplastics and invasive species have primarily focused on their impacts, leaving their combined legacy effects largely unexplored. However, the simultaneous occurrence of microplastic contamination and invasive plant species in agricultural environments is now widely recognized as a significant concern. As a result, understanding the interactions between these two environmental stressors is increasingly important. Microplastics and invasive species can alter the physical, chemical, and biological characteristics of agricultural soils, either alone or together. Meanwhile, microplastics may either exacerbate or alleviate the legacy effects of invasive species. For instance, they can change soil structure, which may enhance the retention or mobility of allelopathic compounds released by invasive species, thereby intensifying negative effects on crops [6]. Conversely, interactions between these stressors could lead to complex synergistic effects, disrupting plant–soil feedback mechanisms and further impacting crop quantity and quality. Ultimately, these adverse effects on crops could threaten food security and agricultural sustainability, particularly in regions where both pollutants are prevalent.

In China, the simultaneous occurrence of alien plant invasions and microplastic contamination in agricultural soils presents a complex challenge with potentially far-reaching consequences [27,28]. The present study aims to investigate the combined effects of the legacy impacts of *S. canadensis* and microplastic contamination on the germination and root development of wheat (*Triticum aestivum* L.), which is one of the most widely cultivated cereal crops in China. *S. canadensis* is a well-known invasive plant in China’s farmland, highly adaptable, high-biomass species competes aggressively with native plants and crops for nutrients and water [16,29]. It also displays exceptional reproductive and dispersal capabilities, even under adverse conditions [23,30]. We hypothesize that the legacy effects of *S. canadensis*, when coupled with soil microplastic contamination, would negatively impact wheat seed germination and seedling growth. Furthermore, we propose that the combined effects of these two stressors would be more detrimental than their individual impacts, potentially leading to significant disruptions in wheat growth. By exploring these interactions, this study seeks to address a critical gap in the current understanding regarding emerging contaminants and biological invasions that jointly affect agricultural ecosystems. Additionally, more studies are needed regarding the seedling’s transcriptomic, metabolomic, genetic, and physiological functions.

## 2. Materials and Methods

### 2.1. Experimental Materials and Design

The experimental soils were collected from the topsoil (0–20 cm) in the green space of Jiangsu University, Zhenjiang, China (32°21′ N, 119°52′ E) in March 2021. The collected soil was sieved through a 2 mm mesh to remove plant debris and large stones and then air-dried for future use. The experimental wheat seeds were procured from the Zhenjiang Jinjia Crop Seed Supply Station, Zhenjiang, China. The experimental *S. canadensis* seeds were collected from farmland in Zhenjiang, where this species had invaded. The experimental seedlings of *S. canadensis* were prepared by growing the seeds in small trays. The polyethylene microplastics with three shapes, which include pellets, fragments, and fibers, were obtained from Zhangmutou Plastic Raw Material Firm, Dongguan, China. The experimental microplastics were precisely hand-cut or sieved to achieve a particle size between 0.5 and 1 mm, with a composition ratio of 3:4:3 for pellets, fragments, and fibers, respectively. For sterilization, the microplastics were immersed in 75% ethanol and then exposed to UV light for enhanced decontamination. They were air-dried for two days to ensure complete evaporation of ethanol and stored at 4 °C until use.

The experiment was conducted in a greenhouse at Jiangsu University. A complete two-factorial design was employed with four treatments and three replicates for each treatment: a control treatment with no soil microplastic contamination or *S. canadensis* invasion (CK), an *S. canadensis* invasion legacy effect treatment (SC), a soil microplastic contamination treatment (MPs), and a combined *S. canadensis* invasion legacy effect with soil microplastic contamination treatment (SC+MPs). For the soil microplastic contamination treatment, sterilized microplastics were mixed into the soil at a precise mass ratio of 0.4%, ensuring uniform distribution throughout the soil matrix to simulate a realistic contamination scenario. For the *S. canadensis* invasion legacy effect treatment, *S. canadensis* was allowed to grow for six months before its removal, thereby creating a legacy effect on the soil. This approach mimicked farmers’ agricultural activities when managing *S. canadensis* invasions of farmland.

In April 2021, 6.0 kg of experimental soil was placed into each pot. The soil’s moisture content was then adjusted to 70% of its maximum water-holding capacity by adding water. For the soil microplastic contamination treatment, sterilized microplastics were mixed with the soil. Further, the soil was processed using the procedure described by Lozano and Rillig [14] to get better results regarding the impact of microplastic contamination in the soil. For the *S. canadensis* invasion legacy effect treatment, 15 seedlings of *S. canadensis* per pot of identical size were transferred into pots after one month of cultivation and watered every two days. After six months of cultivation, the *S. canadensis* seedlings were harvested from all invasion pots.

The experiment officially began on 2 November 2021, with 50 wheat seeds sown in each pot. The pots were then subjected to a controlled growth environment for 30 days, which maintained a constant temperature of 19 ± 1 °C and a humidity level of 76% and the pots were watered every two days.

### 2.2. Seed Germination Rate and Plant Phenotypic Properties Measurement

Throughout the first 12 days of cultivation, the germination of wheat seeds was meticulously monitored and recorded daily. The seed germination rate, the germination potential, and the relative change of germination rate were computed employing the Formulas (1)–(3) [31]. The difference between germination potential and final germination rate is that the previous focuses on the germination rate during the initial days of the experiment, while the latter represents the ultimate germination percentage. The formulas for these are as follows:

(1) $$\mathrm{Germination}\;\mathrm{rate}\;\left(\%\right)=\frac{\mathrm{Number}\;\mathrm{of}\;\mathrm{seeds}\;\mathrm{germinated}\;\mathrm{within}\;X\;\mathrm{days}}{\mathrm{Total}\;\mathrm{number}\;\mathrm{of}\;\mathrm{seeds}\;\mathrm{tested}}\times 100\%$$

where X represents the days of cultivation, and

(2) $$\mathrm{Germination}\;\mathrm{potential}\;\left(\%\right)=\frac{\mathrm{Number}\;\mathrm{of}\;\mathrm{seeds}\;\mathrm{germinated}\;\mathrm{in}\;\mathrm{the}\;\mathrm{first}\;X\;\mathrm{days}}{\mathrm{Total}\;\mathrm{number}\;\mathrm{of}\;\mathrm{seeds}\;\mathrm{tested}}\times 100\%$$

where X is generally 3 days, but in this experiment, wheat seeds did not germinate on the third day, so the sixth day with the fastest germination speed was chosen as the germination potential.

(3) $$\mathrm{Relative}\;\mathrm{change}\;\mathrm{of}\;\mathrm{germination}\;\mathrm{rate}=\frac{Gt-Gc}{Gc}\times 100\%$$

where *Gt* represents the germination rate under treatment conditions, and *Gc* represents the germination rate under control.

On the 30th day of the experiment, both the chlorophyll and leaf nitrogen content of the wheat were measured using a SPAD meter (SPAD-502, Minolta Camera Co., Osaka, Japan). After the measurements, all wheat seedlings were harvested, and the surface soil was rinsed off with clean water. The seedlings were then carefully separated into root and stem sections, and the wheat stalk’s height and the primary root’s length were measured. Then, the wheat root system was scanned using a root scanner, i.e., EPSON Flatbed Scanner Perfection V800 (REGENT Instruments Inc., Quebec City, QC, Canada), to obtain root phenotypic index data, including the root volume, the average root diameter, the root surface area, as well as the number of root tips, branches, and crossovers. Finally, all the samples were placed in a drying oven set at 60 °C and dried until they reached a constant weight. The biomass was then weighed and recorded.

### 2.3. Seedling Vigor Index

Seedling vigor is a measure of the extent of damage that accumulates as viability declines. The damages accumulate in the seeds until the seeds are unable to germinate and eventually die. The seedling vigor (SVI) index was computed employing the Formula (4):

Seedling vigor index = Germination rate × Root length(4)

### 2.4. Statistical Analysis

A two-way analysis of variance (ANOVA) with the least significant difference post-hoc multiple comparison analysis, and a principal component analysis (PCA) with a permutational multivariate ANOVA (PERMANOVA) were employed to test the individual and combined effect of *S. canadensis* invasion legacy effect and soil microplastic contamination on the seed germination potential and the seedling development properties of wheat. Furthermore, redundancy analysis (RDA) was used to investigate plant phenotype and seedling development, and the mantel test was used to analyze the relationship between plant phenotype and seedling height and biomass. In addition, linear regression was used to predict SVI, including root surface area and volume. All of the above analyses were performed using R software (version 4.2.2).

## 3. Results

### 3.1. The Individual and Combined Effect of S. canadensis Invasion Legacy Effects and Soil Microplastic Contamination on Wheat Seed Germination

**Figure 1:**
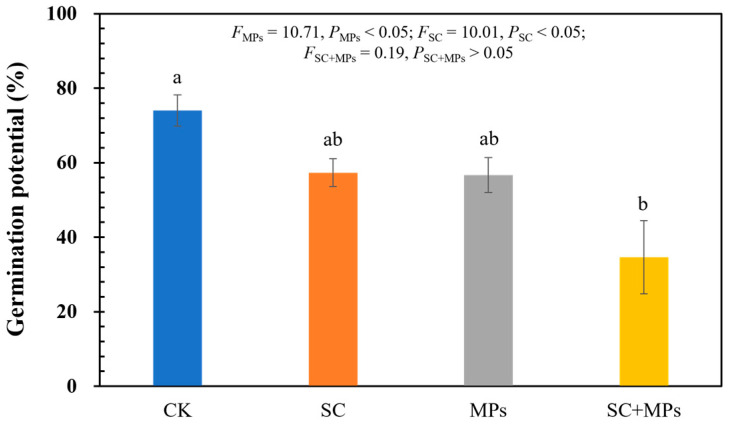
The germination potential of wheat seeds under different treatments. Treatments represent: CK = control treatment with no soil microplastic contamination or *S. canadensis* invasion legacy effect; SC = *S. canadensis* invasion legacy effect treatment; MPs = soil microplastic contamination treatment; SC+MPs = combined *S. canadensis* invasion legacy effect with soil microplastic contamination treatment. The vertical bars on the columns indicate standard errors of the mean (n = 3). Lowercase letters represent the significance of the treatments.

The germination results for wheat seeds revealed that both the invasion legacy effect of *S. canadensis* (SC) and microplastic contamination (MPs) significantly influenced wheat seed germination (Figure 1 and Figure S1). Notable changes in germination rates across different treatment soils became evident from the 4th day after planting, with pronounced effects observed on the 6th day compared to the control treatment (CK). On the 6th day, germination rates of the seeds under either the individual invasion legacy effects of *S. canadensis* or the microplastic contamination treatments decreased significantly, and the combined treatment exhibited an even greater reduction (*p* < 0.05; Figure S1). Furthermore, the germination potential of wheat seeds demonstrated that the *S. canadensis* invasion legacy effect and soil microplastic contamination treatments only induced significant reductions in germination potential independently (all *p* < 0.05). Compared to the control treatment, both the *S. canadensis* invasion legacy effect and soil microplastic contamination treatments diminished wheat seed germination potential by 22.52% and 23.42%, respectively, with the combined treatment exerting the most pronounced inhibitory effect by 53.15% (Figure 1).

### 3.2. The Individual and Combined Effect of S. canadensis Invasion Legacy Effects and Soil Microplastic Contamination on the Development of Wheat Seedlings

**Figure 2:**
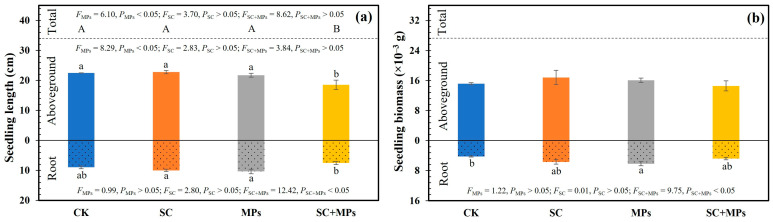
The seedling length (**a**) and biomass (**b**) under different treatments. Different uppercase letters represent significant differences at *p* < 0.05 at the total value, while lowercase letters represent significant differences at *p* < 0.05 at the root and aboveground seedling growth. The vertical bars on the columns indicate standard errors of the mean (n = 3).

**Figure 3:**
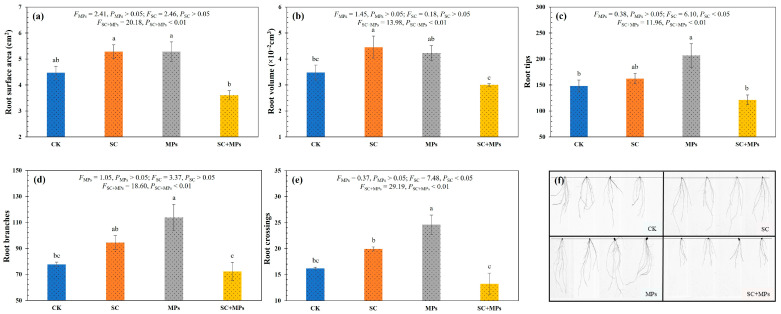
The root surface area (**a**), root volume (**b**), root tips (**c**), root branches (**d**), root crossings (**e**), and rhizosphere scan picture (**f**) under different treatments. Different lowercase letters represent significant differences at *p* < 0.05). The vertical bars on the columns indicate standard errors of the mean (n = 3).

The results showed that the individual and combined treatments of the legacy effects of *S. canadensis* invasion and soil microplastic contamination significantly influenced wheat seedling properties (all *p* < 0.05), except for aboveground biomass (*p* > 0.05) and total biomass (*p* > 0.05). Specifically, the independent application of *S. canadensis* invasion legacy effects or soil microplastic contamination promoted wheat seedling development. In contrast, the combined treatment inhibited seedling development, showing significant deviations from the control treatment (Figure 2 and Figure 3).

The soil microplastic contamination treatment alone significantly induced changes in aboveground length and total length (both *p* < 0.05; Figure 2a). In contrast, the legacy effects of *S. canadensis* invasion alone significantly affected root tips and root crossings (both *p* < 0.05; Figure 3c,f). Meanwhile, the significant combined effects of *S. canadensis* invasion legacy effect and soil microplastic contamination were observed on seedling root length (*p* < 0.01), total length (*p* < 0.05), root biomass (*p* < 0.05), root surface area (*p* < 0.01), root volume (*p* < 0.01), root tips (*p* < 0.01), root branches (*p* < 0.01), and root crossings (*p* < 0.01; Figure 2 and Figure 3). In addition, the combined effects of *S. canadensis* invasion legacy effect and soil microplastic contamination led to a decrease in SPAD and leaf nitrogen, but not significantly (Figure S2).

Compared to the control treatment, the legacy effects of *S. canadensis* invasion and soil microplastic contamination individually altered aboveground length, root length, and total length by 1.09% and −3.57%, 11.31% and 15.45%, and 3.99% and 1.84%, respectively. In contrast, the combined effect decreased aboveground length, root length, and total length by 17.74%, 16.32%, and 17.34%, respectively. In addition, the legacy effects of *S. canadensis* invasion, soil microplastic contamination, and their combined treatment increased root biomass by 33.88%, 45.46%, and 12.14%, respectively (Figure 2).

Additionally, compared to the control treatment, the legacy effects of *S. canadensis* invasion and soil microplastic contamination individually increased root surface area, root volume, root tips, root branches, and root crossings by 18.18% and 18.28%, 28.07% and 21.47%, 9.62% and 39.66%, 21.56% and 46.47%, and 23.02% and 51.89%, respectively. However, the combined treatment decreased these parameters by 19.40%, 13.77%, 18.03%, 7.06%, and 18.32%, respectively (Figure 3).

**Figure 4:**
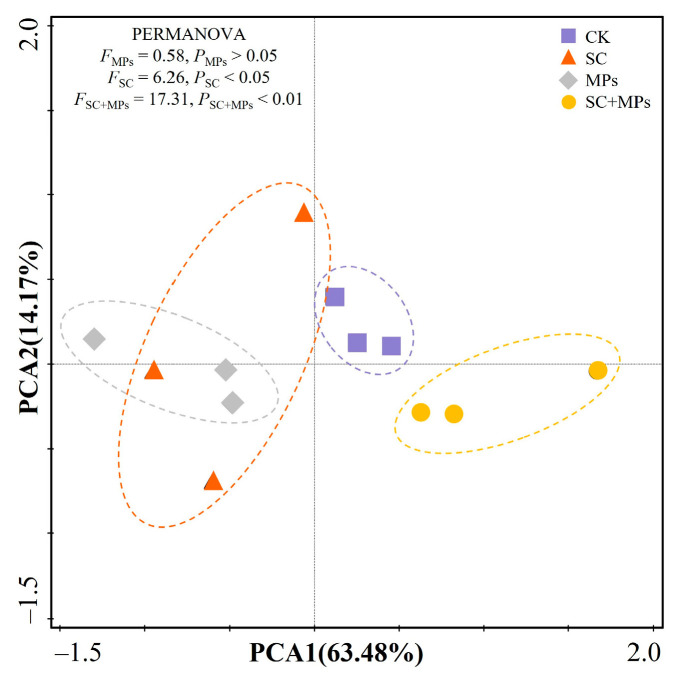
Principal component analysis (PCA) of wheat seedling parameters under different treatments.

The PCA diagram with PERMANOVA revealed that microplastic contamination treatment alone did not have a significant impact, whereas the combined treatment of microplastics and the legacy effect of *S. canadensis* invasion exhibited a statistically significant impact (Figure 4). The PCA1 and PCA2 account for these parameters at 63.48% and 14.17%, respectively. Along the PCA1, these parameters are markedly differentiated; individual treatments involving the legacy effects of *S. canadensis* invasion and microplastic contamination are positioned to the left of the control treatment, while the combined interactive effects are situated to the right.

### 3.3. Effects of Plant Phenotypes on the Length and Biomass of Seedling and Predictors of SVI

**Figure 5:**
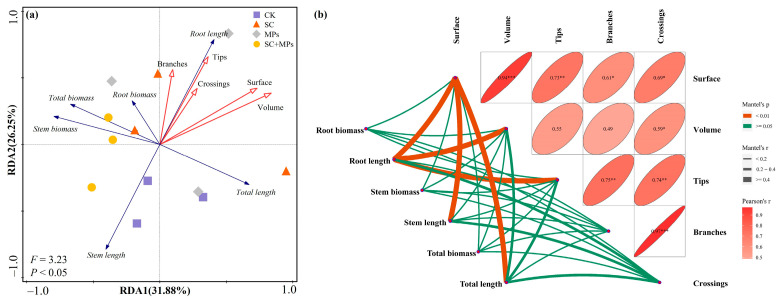
Redundancy analysis of plant phenotype and seedling development (**a**); Mantel test analysis between plant phenotype and seedling development (**b**). The correlation is statistically significant and represented by highly significant with *p* < 0.001 (***), *p* < 0.01 (**), significant with *p* < 0.05 (*), and non-significant.

The RDA results showed that plant phenotype had more influence on the seedling length, a positive influence on the root system, and a negative influence on the aboveground part, but negligible effect on the total length and biomass (Figure 5a).

Mantel test results showed that stem length was significantly correlated with root surface area (R = 0.44, *p* < 0.01), that root length was significantly correlated with root surface area (R = 0.86, *p* < 0.01), root volume (R = 0.52, *p* < 0.01), and root tips (R = 0.60, *p* < 0.01), and that total length was significantly correlated with root surface area (R = 0.65, *p* < 0.01) (Figure 5b).

**Figure 6:**
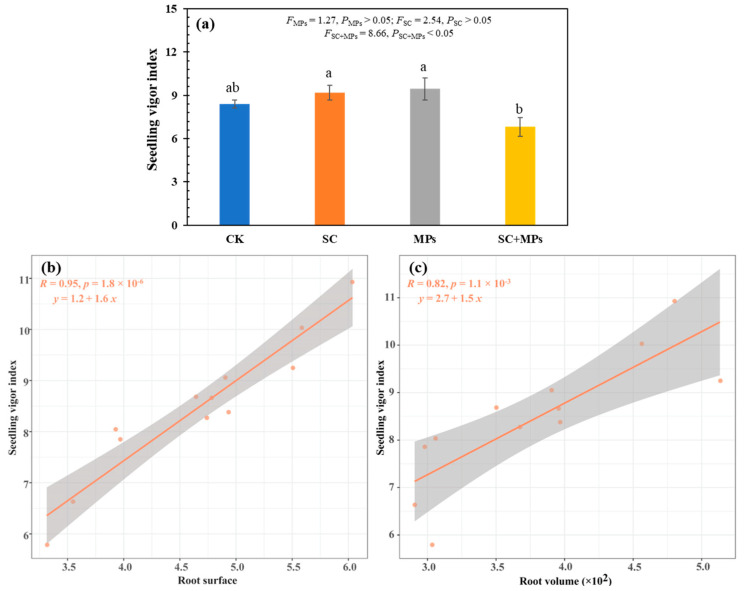
The wheat seedling vigor index under different treatments (**a**). Linear regression between seedling vitality vigor and seedling root surface area (**b**), and linear regression between seedling vigor index and seedling root volume (**c**). Different lowercase letters represent significant differences at *p* < 0.05.

The results of SVI showed that the *S. canadensis* invasion legacy effect and the soil microplastic contamination treatment alone had a slight promotion effect on SVI, but the combined treatment had a significant inhibition effect on it (Figure 6a). In addition, linear regression results showed that SVI increased with an increase in the root surface area and volume (Figure 6b,c). Linear regression analysis between SVI and seedling root surface area revealed that the results for the control, *S. canadensis* invasion legacy effect, and soil microplastic contamination treatments were not statistically significant. However, the combined treatment showed a significant relationship (Figure S3a). Interestingly, there were no significant results in grouping linear regression between the SVI and seedling root volume (Figure S3b).

## 4. Discussion

### 4.1. Impact of Legacy Effects of S. canadensis Invasion and Soil Microplastic Contaminants on Wheat Seed Germination

Since germination potential primarily reflects the speed and vigor of seed germination during the early stages, it stands as a pivotal indicator, gauging the potency of seed germination under different environmental stresses. The results of this study support our hypothesis that both the legacy effects of *S. canadensis* invasion and soil microplastic contamination would influence wheat seed germination potential as well as germination rate (Figure 1 and Figure S1). However, the results indicated that these treatments primarily affect the germination potential rather than reducing the overall germination rate. Moreover, microplastics and invasion legacy effects displayed negative effects on seed germination (*p* < 0.05), while the final germination rate was not significant (*p* > 0.05; Figure S1). This change may result from the altered soil properties caused by residual allelopathic compounds released by *S. canadensis* and the presence of microplastics, which likely disrupt the optimal conditions required for wheat germination, slowing the process without completely inhibiting it. Previous studies have shown that environmental stressors such as allelopathic chemicals and soil pollutants like microplastics could extend germination periods, reduce seedling vigor, and ultimately impact crop productivity [6,32,33]. The findings from this study align with these observations, particularly regarding the negative effects of combined stressors on early plant development. Moreover, the combined treatment of *S. canadensis* invasion legacy effects and soil microplastic contaminants had a more pronounced impact on the wheat germination potential timeline than the individual treatments, highlighting the potential synergistic effects of these environmental stressors. This synergism may be due to the interaction between microplastics and the allelopathic compounds from *S. canadensis*, where microplastics act as carriers, prolonging the presence and mobility of these compounds in the soil, thereby exacerbating their inhibitory effects on wheat germination.

### 4.2. Individual Effects of S. canadensis Invasion Legacy Effects or Soil Microplastic Contaminants on Wheat Seedlings

When applied independently, the distinct positive effects of each stressor suggested that both *S. canadensis* invasion legacy effects and soil microplastic contamination contribute to an enhanced root system and biomass in wheat seedlings while suppressing all these parameters in the interactive treatment effects (Figure 2 and Figure 3). On the one hand, *S. canadensis*, a nitrogen-affinitive species, can enhance soil nitrogen availability by altering the soil properties via the release of allelopathic chemicals in the soil. Studies have shown that the invasion of *S. canadensis* significantly increases soil nitrogen content by modifying the soil physical and chemical properties as well as microbial communities [29,34,35,36,37,38]. Even after the removal of *S. canadensis*, the soil would continue to be altered, creating a nitrogen-rich environment known as the “legacy effects” [39,40,41]. Creating a more nitrogen-rich environment would further enhance the root development and biomass of wheat seedlings. In this study, the *S. canadensis* invasion legacy effect treatment induced higher nitrogen content in the wheat seedling leaf (Figure S2b). Thus, the wheat root development and biomass were promoted under the *S. canadensis* invasion legacy effect treatment (Figure 2 and Figure 3).

On the other hand, microplastic contamination, increasingly recognized as an emerging pollutant in agricultural soils, has shown both beneficial and harmful effects on plant root systems [2,4]. When present independently, microplastics enhanced the root growth of wheat in this study (Figure 2 and Figure 3). This enhancement may be caused by the microplastic-induced reduction in bulk density, which can be translated into improved soil porosity and permeability [37,42]. This allows for better aeration and root respiration [13,42,43,44]. Meanwhile, microplastics can alter the hydrophilic properties of the soil and thus increase water retention [11,24]. This mechanical disturbance likely induces a mild stress response in wheat seedlings, activating their intrinsic repair mechanisms and promoting root development [45,46]. These are supported by previous studies where low concentrations of microplastics triggered a hormetic response—a phenomenon where mild stressors stimulate plant growth and enzymatic activity [37]. However, soil microplastics may exceed the beneficial stress threshold at higher concentrations or in combination with other stressors like *S. canadensis* invasion, which may lead to toxic effects [47,48,49]. Additionally, soil microplastics can serve as a carbon source, increasing the dissolved organic carbon in the soil. This can facilitate nutrient absorption by wheat, resulting in a more developed root system compared to the control treatment [40,50].

### 4.3. Interaction Effects of S. canadensis Invasion Legacy Effects and Soil Microplastic Contaminants on Wheat Seedlings

The legacy effects of *S. canadensis* invasion and soil microplastic contamination showed a clear inhibitory effect on the growth and development of wheat seedlings (Figure 2, Figure 3 and Figure 6). These results suggested a complex interaction between these two environmental stressors, which can alter both the direction and intensity of their effects when present together compared to their independent occurrence. This supports our hypothesis that the combined effects of these stressors are more detrimental than their individual impacts. This finding is consistent with previous studies, which reported that the combination of soil microplastics and invasive species alters soil properties and adversely affects plant growth [51,52]. It might also be due to the blockage caused by microplastics, which cling to the root surface and restrict the nutrients and water mobility to the plants [53]. Additionally, this may be linked to the toxic effects of the compounds released from the combined treatment, which can disrupt metabolism in roots, leading to cellular damage and impaired root development [54].

A notable observation was that soil microplastic contaminants, when interacting with the legacy effects of *S. canadensis* invasion, significantly influence the physiological processes of wheat (Figure 2, Figure 3 and Figure 4). This combined effect appears to disrupt seedling root system development and nutrient acquisition and transport, potentially by altering the soil micro-environment. Such a synergistic interaction might likely alter soil structure and nutrient balance, hindering the seedling nutrient and water uptake and severely impeding seedling health and development. It was observed that the interaction between microplastics and *S. canadensis* legacy effects impaired the structural integrity of the wheat seedling root system. Root length, surface area, volume, tips, branches, and crossings of seedlings were lower under the combined treatment conditions than individual treatments or the control treatment (Figure 3). These morphological changes in the root system can diminish root functionality, reducing the seedling’s ability to absorb adequate nutrients and water [6,55]. The damaged root system can further impair plant roots’ absorption and transport of nutrients and water. In this study, the structural integrity of the wheat seedling root system showed a positive relationship with the seedling height and biomass (Figure 5). These synergistic effects are well supported due to the biomass and seedling vigor leading to the altered metabolic and physiological pathways in wheat seedlings. Meanwhile, it was also observed that the combined effect impaired the photosynthetic capacity of a seedling, as evidenced by decreased SPAD values and leaf nitrogen contents (Figure S2). This reduction in photosynthetic capacity may result from inefficient nutrient transport from damaged root systems to other parts of the seedlings [56,57]. Previous studies have shown that soil microplastics can modify soil structure, affecting root development and resource competition with soil microbes [16,43,58,59]. Additionally, microplastics have been linked to an increased oxidative stress in roots, which could further inhibit root development and impair cellular functions critical for growth and nutrient assimilation [60,61,62]. Moreover, the allelopathic compounds left by *S. canadensis* may amplify these disruptions, further reducing root length, surface area, and overall biomass.

### 4.4. Implication for Agricultural Sustainability and Management

The relationship between SVI, root surface area, and volume reflects important mechanisms of plant access to water and nutrients and the ability to interact with the soil environment [35,54]. An increase in root surface area expands the root’s contact with the soil, improving the plant’s efficiency in water and nutrient absorption (Figure 6b). This enhanced absorption capacity provides seedlings with adequate resources, promoting an increased vigor index. Additionally, the increase in root volume indicates that the overall growth and development of the root system are more robust, strengthening the plant’s mechanical support and providing seedlings with greater survival and competitive advantages under varying environmental conditions (Figure 6c). The synergistic growth of root surface area and volume provides physiological support and accelerates seedling growth by improving nutrient supply and water transport, significantly increasing the vigor index [63]. Thus, the increase in root surface area and volume directly impacts seedling growth and development, highlighting the importance of a robust root structure as a key factor in maintaining a high vigor index [17,41].

The results of this study demonstrated that both soil microplastic contamination and the legacy effects of *S. canadensis* invasion significantly impact wheat germination and seedling development. The combined effects of these two stressors are more detrimental than their individual impacts (Figure 1, Figure 2, Figure 3 and Figure 6). The results of combined treatment processing in grouped linear regression prove this (Figure S3). This disruption is particularly concerning in agricultural contexts, as it directly translates into reduced crop yields and poor plant health. The observed suppressed seedling growth suggest that crops in contaminated soils may experience prolonged stress, ultimately reducing yield and quality. The physiological disruptions, including impaired root architecture and reduced biomass accumulation, emphasize the complex nature of plant responses to multiple stressors. Given the increasing prevalence of microplastics in agricultural soils and the widespread distribution of invasive species like *S. canadensis*, these results underscore an urgent need to consider this complexity when developing agricultural management to mitigate the effects of these emerging ecological and environmental issues.

Agricultural management interventions are urgently needed to reduce soil microplastic contamination and to control invasive species in agricultural landscapes. Traditional soil treatments may not be sufficient to mitigate the combined effects of microplastics and invasive species. Therefore, novel approaches, such as soil remediation techniques targeting microplastic contamination and the allelopathic effects of invasive species, are critical. These interventions could include the development of soil conditioners that restore the soil structure, improve microbial communities, and enhance nutrient cycling, as well as using biodegradable plastic alternatives to reduce future contamination. Additionally, promoting sustainable farming practices, such as minimizing plastic-based agricultural products and implementing biosecurity measures to prevent the spread of invasive species, will be essential for maintaining long-term soil health and food security.

### 4.5. Limitations of the Study

This study has certain limitations that should be acknowledged. Firstly, the experiments of this study were conducted in a controlled laboratory setting, which may not fully capture the complexity of real-world agricultural environments. Factors such as soil heterogeneity, weather variability, and other environmental stressors could influence outcomes in field conditions. Additionally, the study primarily examined short-term effects, potentially overlooking the long-term impacts of *S. canadensis* legacy effects and microplastic contamination on crops. Secondly, while the study focused exclusively on wheat, the findings may not be directly applicable to other crops with different ecological and physiological traits. Further study on a broader range of plant species is necessary to validate these results and even at community levels. Thirdly, the combined effects of *S. canadensis* legacy impacts and microplastics were analyzed using a specific form and concentration of microplastics. The diversity of microplastic types, sizes, and concentrations, as well as interactions with other environmental factors like soil pH and microbial activity, may lead to varying outcomes not captured in this study. Finally, this study primarily assessed phenotypic responses and did not delve deeply into the physiological or molecular mechanisms underlying the observed effects. Future research should focus on exploring these mechanisms, particularly the interactions between allelopathic compounds from *S. canadensis* and microplastic-induced stress, to better understand the pathways through which these stressors impact plant development. Despite these limitations, this study provides valuable insights into the complex interactions between biological invasions and emerging contaminants, laying the groundwork for future research and agricultural management strategies.

## 5. Conclusions

This study revealed that both soil microplastic contaminations and *S. canadensis* invasion legacy effects, acting individually or in combination, significantly affect germination and impair wheat seedling development. The most pronounced impacts on the germination rate of wheat occurred under the combined treatment. Interestingly, while soil microplastic contaminations or *S. canadensis* invasion legacy effects alone can enhance seedling development, this effect was diminished or reversed when these environmental stressors were present together, highlighting the complex interactions between these environmental stressors. Our findings underscore the compounded synergistic negative effects of microplastic contamination and invasive species on agricultural systems. This synergistic interaction indicated that the legacy effects of invasive species and emerging contaminants like microplastics may pose more severe threats to crop health than previously understood. Given the global prevalence of soil microplastic contaminations and the increasing spread of invasive species, these results are crucial for shaping future agricultural management practices. There is an urgent need to address these dual threats through improved soil remediation strategies and stricter invasive species control. Further research should focus on the long-term impacts of such combined environmental stressors on crop yield and food security and explore potential mitigation techniques to minimize their detrimental effects on plant development and ecosystem health.

## Data Availability

All the data will be made available on request.

## Supplementary Materials

The following supporting information can be downloaded at: https://www.mdpi.com/article/10.3390/plants14020181/s1, Figure S1: The wheat seed germination rate (a) and relative change rate of germination rate (b) under different treatments. Figure S2: The SPAD (a), and leaf nitrogen (b) under different treatments. Figure S3: Linear regression between seedling vitality vigor and seedling root surface area (a), and linear regression between seedling vigor index and seedling root volume (b).
