# Supplementary material for: Combined Impact of Canada Goldenrod Invasion and Soil Microplastic Contamination on Seed Germination and Root Development of Wheat: Evaluating the Legacy of Toxicity
Source: Plants (Basel). 2025 Jan 10;14(2):181. doi: 10.3390/plants14020181 (PMC11768274; doi:10.3390/plants14020181)
Supplement: Supplementary file 1 [file plants-14-00181-s001.zip › plants-3290764-supplementary.pdf]

Supporting information

Combined Impact of Canada Goldenrod Invasion and Soil Microplastic Contamination on Seed Germination and Root Development of Wheat: Evaluating the Legacy of Toxicity

Guanlin Li [1,2,†], Yi Tang [1,†], Hongliang Xie [1,3], Babar Iqbal [1,*,‡], Yanjiao Wang [1], Ke Dong [4], Xin Zhao [5], Hyun-Jun Kim [6,*,‡], Daolin Du [7] and Chunwang Xiao [8,*,‡]

[1] School of Environment and Safety Engineering, Jiangsu University, Zhenjiang 212013, China; liguanlin@ujs.edu.cn (G.L.); ty12212021@163.com (Y.T.); hongliangxie1018@163.com (H.X.); 17797313436@163.com (Y.W.)
[2] Jiangsu Collaborative Innovation Center of Technology and Material of Water Treatment, Suzhou University of Science and Technology, Suzhou 215009, China
[3] School of Ecology and Environment, Inner Mongolia University, Hohhot 010021, China
[4] Division of Bio Convergence, Kyonggi University, Suwon 16227, Republic of Korea
[5] Department of Civil and Environmental Engineering, College of Engineering, Seoul National University, Seoul 08826, Republic of Korea; zhaoxin@snu.ac.kr
[6] Department of Forest Resources, Chonnam National University, Gwangju 61186, Republic of Korea
[7] Jingjiang College, Institute of Environment and Ecology, School of Environment and Safety Engineering, School of Emergency Management, School of Agricultural Engineering, Jiangsu University, Zhenjiang 212013, China; ddl@ujs.edu.cn
[8] College of Life and Environmental Sciences, Minzu University of China, Beijing 100081, China
* Correspondence: babar@ujs.edu.cn (B.I.); hjkim0837@jnu.ac.kr (H.-J.K.); cwxiao@muc.edu.cn (C.X.);
Tel.: +86-51188790955 (B.I.); +82-28808743 (H.-J.K.); +86-1068932241 (C.X.)
† These authors contributed equally to this work and shared the first author.
‡ These authors also contributed equally to this work and shared the corresponding author.

Figures: S1-S3

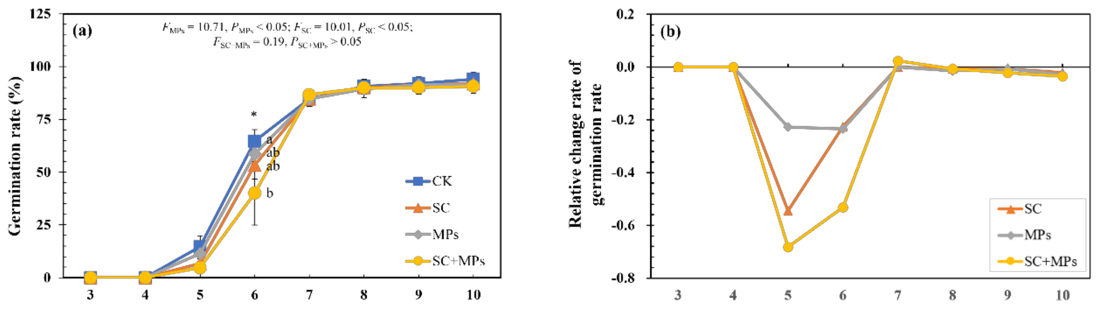

Figure S1 *The wheat seed germination rate (a) and relative change rate of germination rate (b) under different treatments. Treatments represent: CK = Control treatment with no soil microplastic contamination or S. canadensis invasion legacy effect; SC = S. canadensis invasion legacy effect treatment; MPs = soil microplastic contamination treatment; SC+MPs = Combined S. canadensis invasion legacy effect with soil microplastic contamination treatment. The vertical bars on the columns indicate standard errors of the mean (n = 3).*

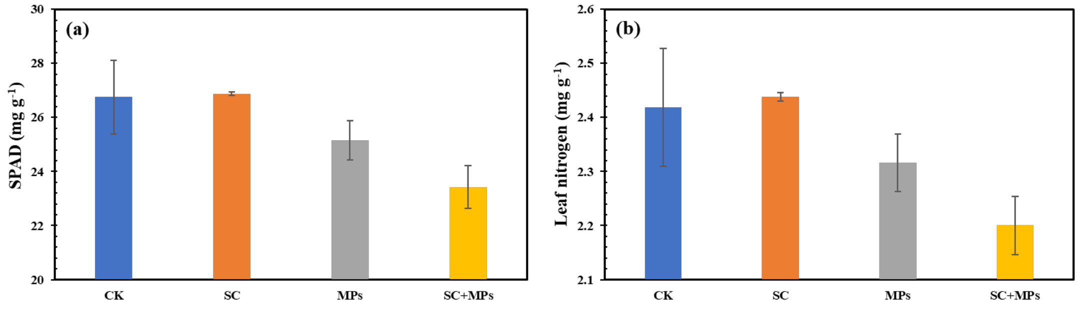

Figure S2 *The SPAD (a), and leaf nitrogen (b) under different treatments. Different lowercase letters represent significant differences at P < 0.05. The vertical bars on the columns indicate standard errors of the mean (n = 3).*

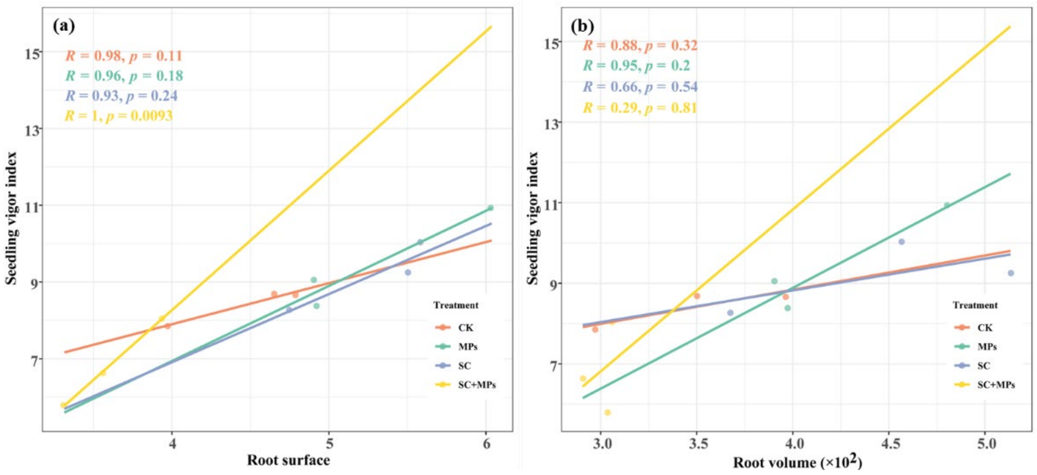

Figure S3 *Linear regression between seedling vitality vigor and seedling root surface area (a), and linear regression between seedling vigor index and seedling root volume (b). Different lowercase letters represent significant differences at P < 0.05. The vertical bars on the columns indicate standard errors of the mean (n = 3).*
